# Genetic Analysis of Genes Related to Tight Junction Function in the Korean Population with Non-Syndromic Hearing Loss

**DOI:** 10.1371/journal.pone.0095646

**Published:** 2014-04-21

**Authors:** Min-A Kim, Ye-Ri Kim, Borum Sagong, Hyun-Ju Cho, Jae Woong Bae, Jeongho Kim, Jinwook Lee, Hong-Joon Park, Jae Young Choi, Kyu-Yup Lee, Un-Kyung Kim

**Affiliations:** 1 Department of Biology, College of Natural Sciences, Kyungpook National University, Daegu, Republic of Korea; 2 School of Life Sciences, KNU Creative BioResearch Group (BK21 plus project), Kyungpook National University, Daegu, Republic of Korea; 3 Soree Ear Clinics, Seoul, Republic of Korea; 4 Department of Otorhinolaryngology, Yonsei University College of Medicine, Seoul, Republic of Korea; 5 Department of Otorhinolaryngology-Head and Neck Surgery, School of Medicine, Kyungpook National University, Daegu, Republic of Korea; University of Chicago, United States of America

## Abstract

Tight junctions (TJs) are essential components of eukaryotic cells, and serve as paracellular barriers and zippers between adjacent tissues. TJs are critical for normal functioning of the organ of Corti, a part of the inner ear that causes loss of sensorineural hearing when damaged. To investigate the relation between genes involved in TJ function and hereditary loss of sensorineural hearing in the Korean population, we selected the *TJP2* and *CLDN14* genes as candidates for gene screening of 135 Korean individuals. The *TJP2* gene, mutation of which causes autosomal dominant non-syndromic hearing loss (ADNSHL), lies at the DFNA51 locus on chromosome 9. The *CLDN14* gene, mutation of which causes autosomal recessive non-syndromic hearing loss (ARNSHL), lies at the DFNB29 locus on chromosome 21. In the present study, we conducted genetic analyses of the *TJP2* and *CLDN14* genes in 87 unrelated patients with ADNSHL and 48 unrelated patients with either ARNSHL or potentially sporadic hearing loss. We identified two pathogenic variations, c.334G>A (p.A112T) and c.3562A>G (p.T1188A), and ten single nucleotide polymorphisms (SNPs) in the *TJP2* gene. We found eight non-pathogenic variations in the *CLDN14* gene. These findings indicate that, whereas mutation of the *TJP2* gene might cause ADNSHL, *CLDN14* is not a major causative gene for ARNSHL in the Korean population studied. Our findings may improve the understanding of the genetic cause of non-syndromic hearing loss in the Korean population.

## Introduction

The organization of eukaryotic cells is established by several junctional proteins that effectively compartmentalize internal multicellular environments that need to operate as specialized functional units. Vertebrate junctional complexes, which are also called intercellular junctions, comprise four components: tight junctions (TJs), adherens junctions, desmosomes, and gap junctions [1].

The specific positioning of TJs at the apical part of the lateral membrane enables them to play critical roles as both zippers that seal intercellular spaces tightly [2] and as barriers that regulate ion- and size-selective paracellular permeability in epithelial cells [35–]. In particular, TJs maintain the concentration of K^+^ between endolymph (high K^+^ concentration) and perilymph (low K^+^ concentration) within the organ of Corti [6]. Among the genes related to the TJ, the *TJP2* and *CLDN14* genes are associated with hereditary hearing loss in humans [7], [8].

The *TJP2* gene, which is located on the long arm of chromosome 9, contains 25 exons and 1,190 amino acids. It is also known as the *ZO2* gene, given that it encodes the zonula occludens protein 2 (ZO2) that belongs to the family of membrane-associated guanylate kinase (MAGUK) homologs [9]. The ZO2 protein, an important TJ associated scaffold molecule, contains several protein binding domains including three PDZ (an acronym combining the first letters of three proteins_PSD95, Dig1, ZO-1) domains, an SRC Homology-3 (SH-3) domain, and a guanylate kinase (GuK)-like domain [101–2]. The PDZ domains can interact directly with the occludin and claudin proteins at TJs [13]. The ZO2 protein might regulate cell differentiation, transformation, and proliferation [14]. This gene is expressed at particularly high levels in membrane-attaching hair cells and supporting cells within the organ of Corti [8]. A previous study of an Israeli population revealed that mutations of the *TJP2* gene led to autosomal dominant non-syndromic hearing loss (ADNSHL). Overexpression of the protein was caused by the inverted genomic duplication of 270-Kb at the DFNA51 locus on human chromosome 9 [8]. Moreover, *TJP2* is associated with familial hypercholanemia, a disease characterized by the malabsorption of fats, itching, and increased levels of bile acid in serum [15].

The *CLDN14* gene comprises three exons and codes for five transcript variants with different 5′UTR sequences that encode the same protein, claudin-14, which is 239 amino acids long and contains four transmembrane domains. Claudin-14 is a member of the claudin family, which includes the most important transmembrane components of TJs in the organ of Corti [16]. It plays a key role as a cation-restrictive barrier, especially for potassium, because tight junctions are regulators of extracellular ion concentrations more than intracellular ion concentrations [17]. In humans, mutations of the *CLDN14* gene at DFNB29 locus on chromosome 21 lead to autosomal recessive non-syndromic hearing loss (ARNSHL) [7]. The phenotype of humans with this mutation resembles that of *Cldn14^−/−^* mice insofar as *Cldn14^−/−^* homozygotes display profound hearing loss with no vestibular dysfunction and heterozygotes (*Cldn14^+/−^*) have normal hearing. The profound hearing loss in *Cldn14^−/−^* mice results from loss of outer hair cells in the cochlea [17]. Six mutations in the *CLDN14* gene–including a frameshift mutation, a nonsense mutation, and four missense mutations–have been reported in Caucasian populations [7], [18], [19].

Although several genetic and functional studies of the *TJP2* and *CLDN14* genes have suggested that mutations of these genes were pathogenic in hereditary hearing loss in humans, the roles of mutations of these genes in contributing to hearing loss in East Asian populations, including people of Korean ancestry, have never been reported. To address this deficiency, we conducted screens for both genes in unrelated patients diagnosed with non-syndromic hearing loss (NSHL).

## Materials and Methods

### Subjects

We analyzed 135 unrelated Korean patients, 87 patients of whom had ADNSHL and 48 of whom had ARNSHL or possible sporadic, bilateral sensorineural hearing loss. All patients were recruited from Kyungpook National University Hospital, Daegu; Yonsei University Health System Hospital, Seoul; and Soree Ear Clinic, Seoul, Korea. Patients with a history of environmental factors such as ear infection or fever and other syndromic hearing loss deduced by either clinical questionnaires or physical examinations were excluded from the study. For the control group, we used 100 unrelated Korean subjects with normal hearing, as determined using audiological tests that included pure-tone audiometry (PTA). Written informed consent was obtained from all participants, and approved by the Institutional Review Board (IRB) of Kyungpook National University Hospital and Severance Hospital, Yonsei University College of Medicine.

### Genetic Analysis

Genomic DNA was extracted from peripheral venous blood using a FlexiGene DNA extraction kit (QIAGEN, Hilden, Germany). Mutation screening was performed by direct sequencing of the coding regions of the *TJP2* (OMIM 607709; NM 004817.3; NP 004808.2) and *CLDN14* (OMIM 605608; NM 144492.2; NP 652763.1) genes. Primers for polymerase chain reaction (PCR) amplification and sequencing of the *TJP2* and *CLDN14* genes were designed using Primer3 (http://frodo.wi.mit.edu/). First, amplification of the exon and exon-intron boundaries was performed by PCR using h-Taq polymerase (Solgent, Daejeon, Korea). The PCR product was then digested with exonuclease I (USB, Cleveland, OH, USA) and shrimp alkaline phosphatase (USB, Cleveland, OH, USA) to remove unincorporated nucleotides and primers. We conducted direct sequencing using a BigDye Terminator v3.1 Cycle Sequencing Kit (Applied Biosystems, Foster City, CA, USA). Following ethanol precipitation, the DNA pellets were resuspended in 10 µl of Hi-Di formamide (Applied Biosystems, Foster City, CA, USA). Resuspended PCR products were sequenced using a 3130*xl* genetic analyzer (Applied Biosystems, Foster City, CA, USA), and the data were analyzed using Sequencing Analysis v5.2 (Applied Biosystems, Foster City, CA, USA) and Chromas Pro v1.5 (Technelysium Pty Ltd., Tewantin, QLD, Australia). The sequencing data were then compared with the reference sequences of the *TJP2* and *CLDN14* genes in the NCBI database (http://www.ncbi.nlm.nih.gov/). The dbSNP (http://www.ncbi.nlm.nih.gov/snp/) and 1000 genomes databases (http://www.1000genomes.org/) were used to assess the novelty and likely pathogenicity of the variations detected in the *TJP2* and *CLDN14* genes. CLC Sequence Viewer v6.0.1 software (CLC Bio, Aarhus, Denmark) was used to determine the level of conservation of protein sequences between humans and selected vertebrates.

### Protein Modeling

The structure of human *TJP2* is unknown, however, the first PDZ domain is 97% identical with that of mouse *TJP2*. This domain can be found in PDB-file 2CSJ and contains position 112. We used this experimentally solved protein structure to analyze mutation p.A112T.

## Results

In order to examine the relation between the variations in genes coding for TJ proteins and genetic causes of ADNSHL and ARNSHL in the Korean population, we selected the *TJP2* and *CLDN14* genes, and screened all their exons and exon-intron boundaries. We identified genetic variations in each of the *TJP2* (Table 1) and *CLDN14* (Table 2) genes.

**Table 1:** SNPs of the *TJP2* gene identified in this study.

Location	Nucleotide change	Amino acid change	Heterozygote	Homozygote	SNP ID
Exon 8	c.375C>T	p.A125A	6	0	rs181450555
	c.382C>A	p.Q128K	7	0	rs41305539
Exon 10	c.1137A>G	p.L379L	8	0	rs17062695
Exon 12	c.1446C>A	p.D486E	8	71	rs2309428
Exon 17	c.2004G>A	p.M668I	1	0	rs34774441
	c.2081G>A	p.G694E	1	0	rs201366118
Exon 19	c.2326T>C*	p.L776L	1	0	This study
Exon 22a	c.2715C>T	p.T905T	36	9	rs2282336
	c.2727G>A	p.A909A	35	9	rs2095876
Exon 25	c.3558G>T	p.R1186R	1	0	rs145112366

*Novel variation of *TJP2* gene identified in this study.

**Table 2:** SNPs of the *CLDN14* gene identified in this study.

Location	Nucleotide change	Heterozygote	Homozygote	SNP ID
Exon 4	c.-769G>A*	1	0	This study
	c.-480A>G	4	1	rs219742
	c.-454G>C	4	1	rs188733
Intron 4	c.-271-83delG	14	0	rs11365554
	c.-271-24C>T	3	1	rs219747
Exon 5	c.-179G>A	1	1	rs73902533
	c.-82+155C>T	1	1	rs55828480
Intron 5	c.-82+171T>C	5	6	rs2850110

*Novel variation of *CLDN14* gene identified in this study.

### Variations of the *TJP2* Gene

**Figure 1:**
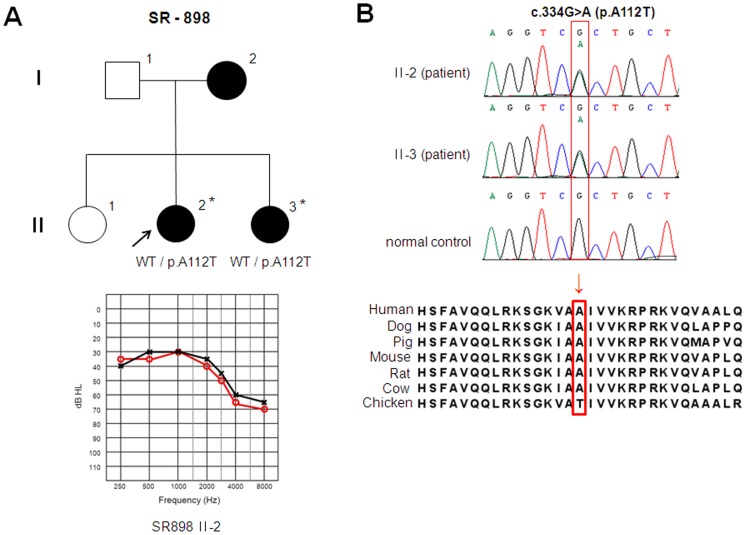
Clinical and genetic information about members of the SR-898 family with the p.A112T mutation in the *TJP2* gene. (A) Top panels show the pedigrees and genotypes of the SR-898 family. The arrow indicates the proband (II-2), and filled symbols represent affected individuals in the family. Asterisks denote individuals screened in this study. The bottom panel is the pure tone audiogram associated with the proband (II-2). The red (circles) and black (crosses) lines represent unmasked air conduction thresholds for the right and left ears, respectively. (B) The top panel shows partial DNA sequences of the *TJP2* gene that indicate the genotypes of patients and normal controls. The position of a changed nucleotide is indicated by a square. The bottom panel shows the evolutionary conservation of amino acids in the *TJP2* sequence, including alanine 112, in vertebrate species. The arrow and box designate the location of the mutation.

**Figure 2:**
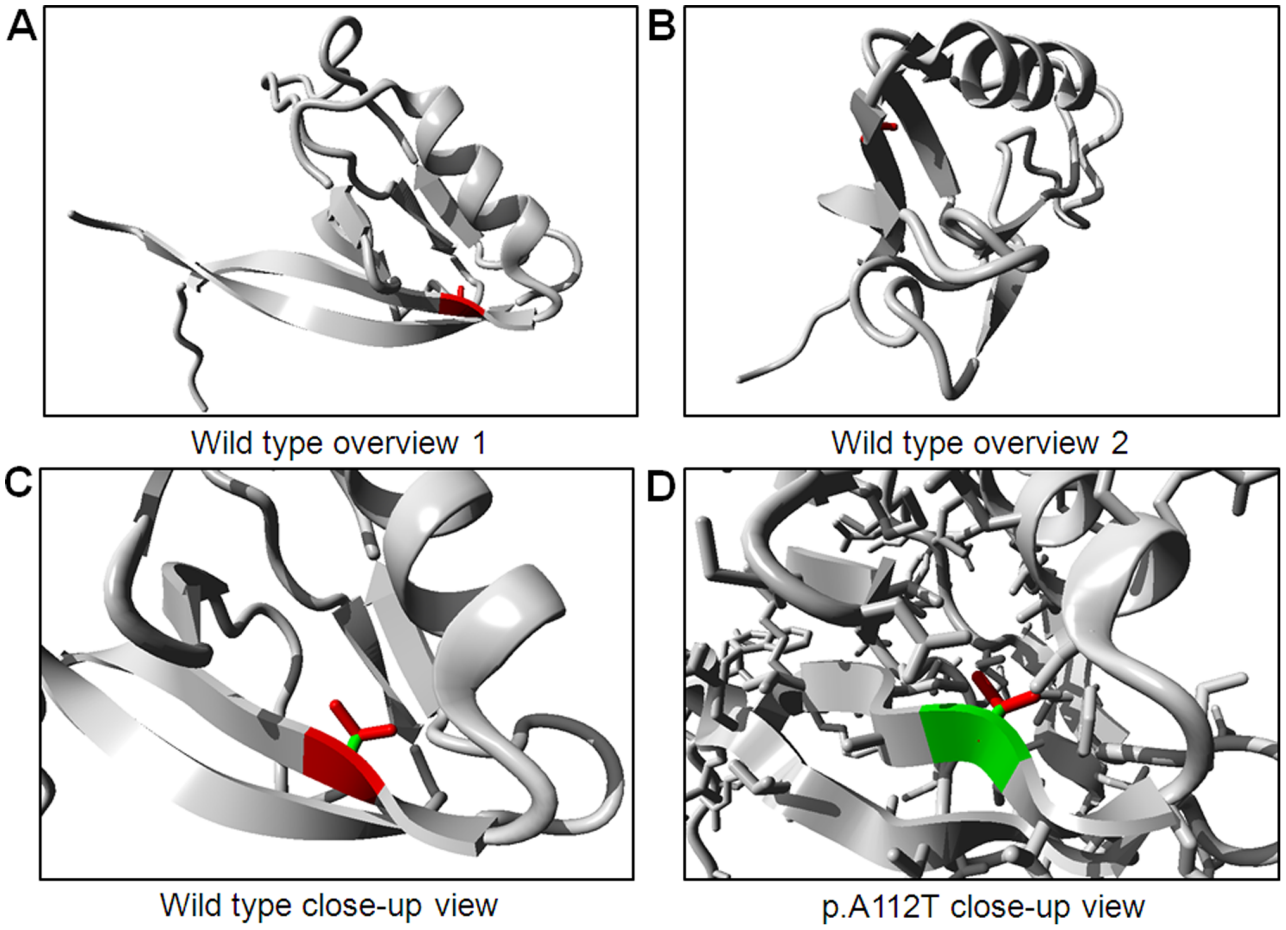
Protein structures of the PDZ 1 domain, including protein position 112 in the *TJP2* protein. (A, B) Overview of the wild type (p.A112). (C, D) Differences between the alanine (C) and threonine (D) at amino acid position 112.

Variations in the *TJP2* gene were evaluated in 87 unrelated Korean patients with ADNSHL. We identified two variations, the c.334G>A (p.A112T) and c.3562A>G (p.T1188A), which are predicted to affect normal hearing. The single-nucleotide change of guanine to adenine at nucleotide position 334 (c.334G>A) was detected in only one patient, II-2 of the SR-898 family, in a heterozygous state. An audiogram of II-2, as diagnosed by the PTA test, shows moderate hearing loss. Another affected member of the SR-898 family, II-3, also retained c.334G>A variation in the heterozygous state (Figure 1A). In addition, none of the 100 unrelated individuals with normal hearing had this variation. This variation leads to the substitution of alanine, an apolar amino acid, by threonine, a polar and slightly bigger amino acid, at position 112 (p.A112T). Alanine 112 is located in the first PDZ domain of ZO2, the sequence of which is conserved in vertebrates such as dog, pig, mouse, rat, cow, and chicken (Figure 1B). We also studied the *TJP2* structure models to establish the molecular differences between wild type (p.A112) and mutant type (p.T112). We found that introduction of a slightly bigger residue at position 112 might destabilize the local structure which can affect the interaction surface which is necessary for the function of the PDZ domain. Besides that, the introduction of a polar side chain might affect correct folding (Figure 2).

**Figure 3:**
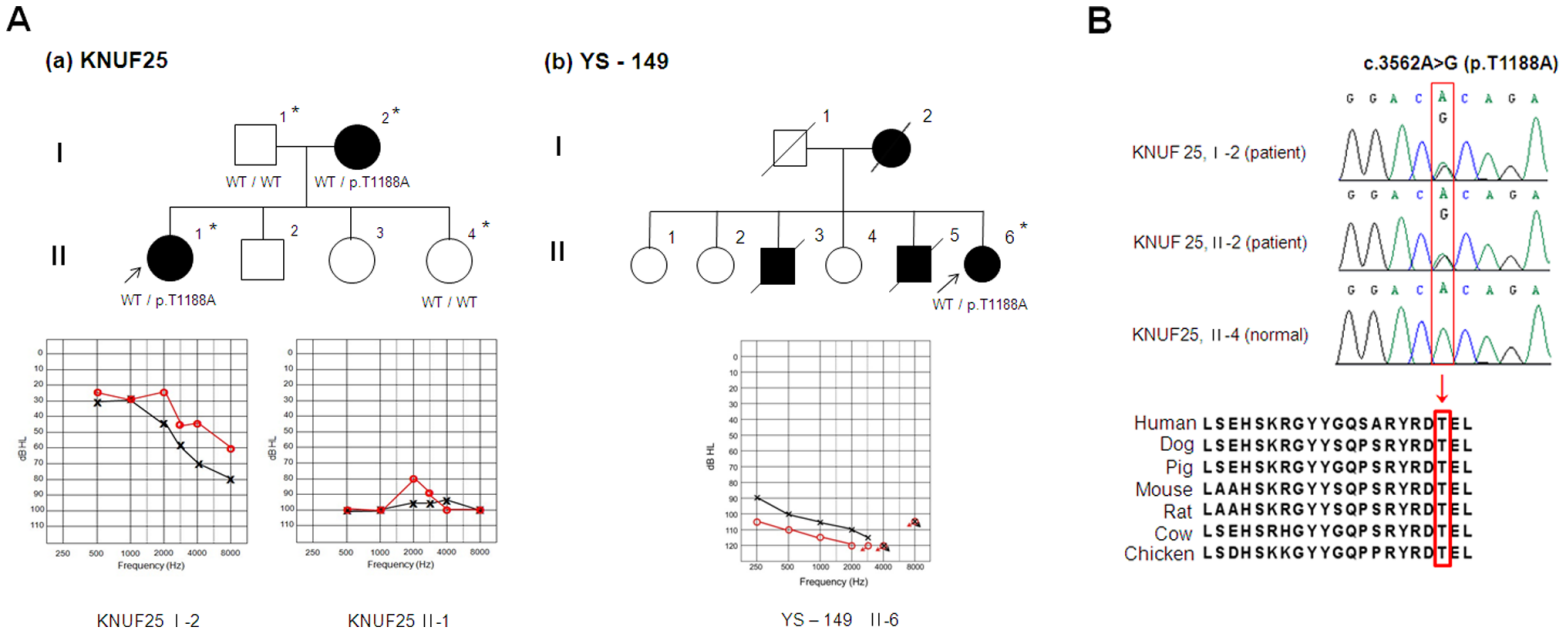
Clinical and genetic information relevant to the KNUF25 (a) and SY-149 (b) families with the p.T1188A mutation of the *TJP2* gene. (A) The top panel shows the pedigree and genotype of each family. The arrows indicate the proband (KNUF25 II-1, YS-149 II-6), and filled symbols represent affected individuals from these families. The asterisks denote individuals screened in this study. The bottom panel shows pure tone audiograms of affected individuals (KNUF25 II-1, KNUF25 I-2, YS-149 II-6). The red (circles) and black (crosses) lines represent unmasked air conduction thresholds for the right and left ears, respectively. (B) The top panel shows partial DNA sequences of the *TJP2* gene with the genotypes of affected and unaffected individuals within the family. The position of a changed nucleotide is indicated by a square. The bottom panel indicates the evolutionary conservation of amino acids, including threonine 1188, in vertebrate species. The arrow and box designate the location of the mutation.

The c.3562A>G variation that we predicted to be pathogenic was detected in two patients, II-1 of the KNUF25 family and II-6 of the YS-149 family, both in the heterozygous state (Figure 3). Figure 3A shows the pedigree of each family and the pure-tone audiograms of the patients. The variant causes a transition of adenine to guanine at nucleotide position 3562 (c.3562A>G), which results in the substitution of the polar amino acid threonine by the apolar amino acid alanine at amino acid position 1188 (p.T1188A). The threonine at this position is highly evolutionary conserved among the vertebrates studied (Figure 3B). Members of the KNUF25 family show a dominant inheritance pattern (Figure 3A). Screening of the *TJP2* gene with regard to c.3562A>G in other members (I-1, I-2, II-4) of the family revealed its presence only in the affected individual (I-2) and its absence in unaffected individuals (I-1 and II-4) of the family. This result indicates that the c.3562A>G variation co-segregates with hearing loss in the KNUF25 family. The PTA test revealed slightly different characteristics of the hearing loss observed in I-2 and II-1. Whereas an audiogram of I-2 shows a downward slope and suggests severe hearing loss at high frequencies, an audiogram of II-1 shows a flat profile (no slope) and profound hearing loss at all of the frequencies tested. In the YS-149 family, the c.3562A>G variation was identified in the II-6 patient (Figure 3A-b). Because the other affected family members passed away, we were unable to further study the loss of hearing in this family. An audiogram of II-6 in the YS-149 family shows a flat profile (no slope) and profound hearing loss at all of the frequencies tested. Additionally, this variation did not exist in 100 unrelated individuals with normal hearing.

Analysis of the *TJP2* gene also revealed ten SNPs, four non-synonymous variations and six synonymous variations, including a novel variation (Table 1). Among them, c.2081G>A was detected in only one patient. This variant involves a transition from a guanine to an adenine nucleotide at position 2081, which causes a glycine to glutamic acid substitution at amino acid position 694 (p.G694E). Because this variation showed a very low global minor allele frequency, with a score of 0.0005, the dbSNP of NCBI suggested that it has a deleterious influence on normal hearing. However, screening of 100 unrelated individuals with normal hearing identified an individual with normal hearing who carried this variation in heterozygous state. This observation enables us to eliminate this variation as a possible cause of hearing loss in the Korean population.

### Variations of the *CLDN14* Gene

Screening of the *CLDN14* gene revealed eight variations in 48 unrelated Korean patients with ARNSHL (Table 2). No variation was identified in exon or in exon-intron boundary of the gene. The detected variations included four variations in the 5′UTRs (including a novel variation) and four intronic variations. However variations observed were not expected to be causative for the disease.

## Discussion

Hearing loss is one of the most common of sensorineural disorders [20]. Among the various types of hearing loss, sensorineural hearing loss is caused by damage to the inner ear. Approximately half of all cases of congenital hearing loss have a genetic basis, including the 35% of all cases that are categorized as non-syndromic [21].

In the inner ear, TJs are essential for the maintenance of compartmentalization and modulation of intercellular permeability [25–], both of which are required for normal functioning of the organ of Corti. We selected the *TJP2* and *CLDN14* genes, each of which encodes a key protein of TJs, to identify mutations responsible for congenital autosomal dominant and recessive NSHL. Screening of the *TJP2* and *CLDN14* genes in Korean individuals with NSHL enabled us to detect 18 and 10 variations, respectively. Among the variations identified in the *TJP2* gene, the variations expected to be pathogenic for NSHL in humans were the c.334G>A (p.A112T) and c.3562A>G (p.T1188A) variants.

The c.334G>A (p.A112T) variation was identified in only one family of the Korean population studied. This variation was previously reported as an SNP in the NCBI database. However the very limited frequency of this variation (0.001) in dbSNP of NCBI led us to anticipate that the c.334G>A (p.A112T) variation might be pathogenic. Screening of affected members (II-2 and II-3) of the SR-898 family suggested that the c.334A mutant allele might be associated with hearing loss in the SR-898 family (Figure 1A). The pure tone audiogram of II-2 suggested moderate to moderately severe hearing loss, which was especially evident at high sound frequencies (Figure 1A). Moreover, we hypothesized that the p.A112T variation may affect binding affinity of ZO2 protein because of its position in the first PDZ domain, which is an essential protein-binding domain. The results of protein modeling revealed the slightly bigger side chain of p.A112T mutation does not fit in the core of the ZO2 protein, and it supported our hypothesis.

In the current study, the c.3562A>G (p.T1188A) variation is situated within the type-I PDZ binding motif TEL, which is highly conserved among vertebrate species. Among the samples screened for the *TJP2* gene, the three individuals with hearing loss who carried the c.3562A>G (p.T1188A) variation were I-2 (age, 62 years) and II-1 (age, 37 years) of the KNUF25 family and II-6 (age, 57 years) of the YS-149 family. Audiograms were recorded for all of the patients who carried this variation (Figure 3). Audiograms from these three affected individuals did not suggest a correlation between the age of the affected members and extent of their hearing loss. For instance, although similar in age, a 62-year-old affected member of the KNUF25 family had substantially better hearing (particularly overall frequencies) than a 57-year-old affected member of the YS-149 family. Moreover, I-2 of the KNUF25 family has considerably better hearing than her daughter, who had profound hearing loss (Figure 3A). The 1000 genomes database using the PolyPhen and SIFT suggested further evidence that the c.3562A>G variation is pathological. Finally, we considered two variations in the *TPJ2* gene, c.334G>A (p.A112T) and c.3562A>G (p.T1188A), as being pathogenic and causally linked to ADNSHL in the Korean population.

We further proposed that synonymous variations with allele frequency of 0.011, such as c.2326T>C (p.L776L) and c.3558G>T (p.R1186R), are located within splicing enhancer sites. Splicing enhancer sites play key roles in the splicing of pre-mRNA into messenger RNA (mRNA). The c.2326T>C (p.L776L) variation, which is reported here for the first time, and the c.3558G>T (p.R1186R) variation were each found in only one proband (Table 1). We investigated whether the c.2326T>C (p.L776L) and c.3558G>T (p.R1186R) variations affected the ESE sequence of which a change in a single nucleotide in the *TJP2* gene would affect the accurate splicing of pre-mRNA into mRNA. However, these variations were not positioned within ESE sequences of the *TJP2* gene. We concluded that only two of the variations detected in the *TJP2* gene are pathogenic and associated with ADNSHL in the Korean population studied.

The second candidate gene studied here, *CLDN14*, was causally linked to ARNSHL in humans [7]. Various pathogenic mutations, such as the c.167G>A (p.W56*), c.242G>A (p.R81H), c.254T>A (p.V85D), c.301G>A (p.G101R), and c.694G>A (p.G232R) have been linked with ARNSHL in Caucasian populations [7], [19]. The present study identified none of these mutations in the Korean population diagnosed with either ARNSHL or possibly sporadic hearing loss. Variations identified in *CLDN14* showed 8 single nucleotide variations in 48 unrelated Korean patients (Table 2). However, none of the variation would affect the function of protein since all of them localized to the CDS region or a splicing site. The *CLDN14* variations positioned at 5′UTR might be associated to the activity of promoter, nevertheless, the previous study reported that c.-480A>G, c.-454C>G, and c.-179G>A variations were not expected to be causative for the disease [18]. Therefore, we consider the c.-769A>G variation first discovered in this study as SNP, and conclude that the *CLDN14* variations identified in the present study are non-pathogenic with regard to ARNSHL in the Korean population studied.

In summary, we report the first genetic analysis of two genes related to TJ function with respect to the incidence of autosomal-dominant and autosomal-recessive NSHL in the Korean population. Our findings suggest that whereas two variations in the *TJP2* gene are casually linked to hearing loss, variations in the *CLDN14* gene do not make a major contribution to the etiology of hearing loss in Korean patients. These results may help to guide future studies for the genetic basis of hearing loss. For instance, further investigations may involve other genes that encode TJ proteins. One such gene, the *TRIC* gene, encodes tricellulin and is known to cause NSHL (DFNB49) [22]. Additional investigation is needed to provide the fundamental information required for an accurate prediction of the genetic cause of the patients diagnosed with NSHL in the Korean population.
